# Pyroligneous Acid in Ulcers and Gangrene

**Published:** 1830

**Authors:** 


21. *Pyroligneous Acid in ulcers and gangrene.* In the last Number of the highly respectable periodical just quoted, Dr. Simons of Charleston, S. C. has inserted a short practical paper, on the use of pyroligneous acid in ulcers of a bad character and in gangrene, from which it appears to be a remedy of considerable power. The following are his remarks on the manner of using it:

There are two kinds of pyroligneous acid found in the apothecary shops; one is transparent, and when agitated, shows small crystals floating in it; the other is dark and smoky; both have the empyreumatic odour. The former is the kind I use, and is by far the best.

When I first used this acid, I diluted it with six times its quantity of water; but since I have employed it diluted with equal parts of water, gradually diluting as the sore assumes a healthy appearance, until it becomes as weak as one-twenty-fourth. It should always create a smarting sensation. The manner of applying it is to put over the ulcer some lint, which is to be kept constantly wet, and changed two or three times during the day, according to circumstances.—

The ulcer uitimately assumes red granulations resembling the inside of the pomegranate. If the acid be too strong, it will make it turn white, and assume the appearance of a slough.

